# The off-target effect of loratadine triggers autophagy-mediated apoptosis in lung adenocarcinoma cells by deactivating JNK, p38, and STAT3 signaling through both PP2A-dependent and independent pathways

**DOI:** 10.3892/ijmm.2025.5495

**Published:** 2025-01-29

**Authors:** Ming-Hsien Chien, Wen-Yueh Hung, Tsung-Ching Lai, Ching Han Tsai, Kai-Ling Lee, Feng-Koo Hsieh, Wei-Jiunn Lee, Jer-Hwa Chang

**Affiliations:** 1 Graduate Institute of Clinical Medicine, College of Medicine, Taipei Medical University, Taipei 11031, Taiwan, R.O.C.; 2 Pulmonary Research Center, Wan Fang Hospital, Taipei Medical University, Taipei 11696, Taiwan, R.O.C.; 3 TMU Research Center of Cancer Translational Medicine, Taipei Medical University, Taipei 11031, Taiwan, R.O.C.; 4 Traditional Herbal Medicine Research Center, Taipei Medical University Hospital, Taipei 11031, Taiwan, R.O.C.; 5 Division of Pulmonary Medicine, Department of Internal Medicine, Wan Fang Hospital, Taipei Medical University, Taipei 11696, Taiwan, R.O.C.; 6 Division of Pulmonary Medicine, Department of Internal Medicine, Taipei Medical University Hospital, Taipei 11031, Taiwan, R.O.C.; 7 The Genome Engineering and Stem Cell Center, School of Medicine, Washington University, St. Louis, MO 63105, USA; 8 Department of Medical Education and Research, Wan Fang Hospital, Taipei Medical University, Taipei 11696, Taiwan, R.O.C.; 9 Department of Urology, School of Medicine, College of Medicine, Taipei Medical University, Taipei 11031, Taiwan, R.O.C.; 10 School of Respiratory Therapy, College of Medicine, Taipei Medical University, Taipei 11031, Taiwan, R.O.C.

**Keywords:** loratadine, autophagy, apoptosis, protein phosphatase 2A, lung adenocarcinoma

## Abstract

Lung adenocarcinoma (LUAD) is a typical inflammation-associated cancer, and anti-inflammatory medications can be valuable in cancer therapy. Loratadine, a histamine receptor H1 (HRH1) antagonist, shows both anti-inflammatory and anticancer properties. The present study aimed to evaluate impacts of loratadine on LUAD cells as well as in a LUAD xenograft mouse model, and explore underlying mechanisms. Mechanistic investigations were conducted through using western blotting, flow cytometry, immunohistochemistry, acridine orange staining, TUNEL assays, and *in silico* analyses of loratadine-modulated genes in LUAD specimens. It was observed that loratadine inhibited LUAD cell proliferation and colony formation by inducing autophagy-mediated apoptotic cell death independently of HRH1. In a LUAD xenograft model, loratadine decreased tumor proliferation and angiogenesis while enhancing autophagy and apoptosis. Mechanistically, loratadine induced protein phosphatase 2A (PP2A) activation to deactivate c-Jun N-terminal kinase (JNK)1/2 and p38 in H23 and PC9 LUAD cells. Additionally, loratadine inhibited signal transducer and activator of transcription 3 (STAT3) activation via a PP2A-independent pathway. Furthermore, the combination of loratadine with inhibitors for JNK, p38 and STAT3 all enhanced proliferation inhibition of loratadine alone in both cell lines. In the clinic, patients with LUAD expressing high PP2A had favorable prognoses. The present study suggests that loratadine can be used as a PP2A activator for LUAD treatment, and the combination of repurposing loratadine with inhibitors of STAT3, JNK and p38 would be an effectively strategy for inhibiting LUAD growth.

## Introduction

Lung cancer ranks among the most frequently diagnosed cancers worldwide, affecting over 2 million patients each year (1). A total of ~90% of these patients are diagnosed with non-small cell lung cancer (NSCLC). Lung adenocarcinoma (LUAD) is the predominant subtype of NSCLC, accounting for ~40% of all lung cancer cases. Notably, the epidermal growth factor receptor (EGFR) mutation is the most prevalent oncogenic mutation in LUAD, particularly among individuals of East Asian descent, women and non-smokers (2). At present, the primary approach for treating NSCLC revolves around identifying targetable driver mutations and immune checkpoints. However, patients who do not possess these characteristics are left with limited options, such as chemotherapy and radiotherapy, but with severe side effects and unpredictable outcomes. Therefore, the pursuit of more-effective treatments for patients lacking these features remains a paramount concern (3).

The repurposing of well-characterized and well-tolerated drugs for cancer therapy has emerged as an appealing alternative to the lengthy and expensive process of new drug development. In recent years, there has been a growing trend of repurposing non-anticancer medications for treating cancers, such as the antidiabetic drugs, metformin and thiazolidinediones, for treating various cancers (4) and the antibiotic, nitroxoline, for treating pancreatic cancer (5). Repurposing offers the potential to discover novel treatments for diseases more cost-effectively and with shorter development timelines. This is especially valuable when preclinical safety data are already available, allowing for the swift evaluation of new therapeutic applications in clinical trials (6).

Tumors maintain an inflammatory microenvironment, and anti-inflammatory medications have demonstrated potential in cancer therapy. Loratadine and its primary metabolite, desloratadine, are potent antagonists of the human histamine receptor H1 (HRH1) and were originally intended to treat allergies and allergic rhinitis (7). Previously, multiple studies reported improved survival associated with the use of loratadine or desloratadine in patients with melanomas (8) and breast cancer (9). In addition to those cancer types, loratadine and desloratadine also showed consistent associations with improved prognoses, especially in other immunogenic tumors, including gastric, colorectal, pancreatic, lung, prostate, kidney and bladder cancers, as well as the non-solid tumor, Hodgkin lymphoma (10). Apart from clinical assessments of loratadine's impact on the prognosis of patients with cancer, several *in vitro* and *in vivo* studies demonstrated the anticancer potential of loratadine and other H1-antihistamines on various cancer cells. For instance, Chen *et al* (11) indicated that the combination of thioridazine and loratadine displayed favorable anti-gastrointestinal cancer effects by inducing cell apoptosis (11). Desloratadine was shown to inhibit the growth and invasion of bladder cancer cells by suppressing the epithelial-to-mesenchymal transition and interleukin-6 expression (12). Additionally, another H1-antihistamine, terfenadine, was reported to induce apoptosis and autophagy in melanoma cells (13). In contrast to those tumor types, the anticancer potential and the underlying mechanisms of loratadine against NSCLC remain unknown. Transforming growth factor β-activated kinase 1 (TAK1) acts as an upstream kinase for both NF-κB and c-Jun N-terminal kinase (JNK)-activator protein-1 (AP-1) signaling pathways (14) and has been implicated in the anti-inflammatory effects of loratadine (15). These signaling pathways have also been associated with poor prognosis, advanced clinical stages and metastasis in NSCLC (16), indicating that loratadine may hold therapeutic potential for NSCLC treatment.

The current study explored loratadine's potential anticancer properties in various LUAD cell lines. These cell lines carry either the wild-type (WT) or mutant EGFR. The study delved into the mechanisms underlying these effects in both *in vitro* experiments and a subcutaneous xenograft model. Our findings indicated that loratadine effectively reduced cell viability and proliferation in various LUAD cell lines, both *in vitro* and *in vivo*, suggesting its potential as an anticancer agent for lung cancer therapy. Additionally, it was observed that loratadine promoted autophagy-mediated apoptotic cell death by suppressing the activation of signal transducer and activator of transcription 3 (STAT3), JNK and p38 through phosphatase 2A (PP2A)-dependent or -independent mechanisms.

## Materials and methods

### Data collection from bioinformatics analyses

The PRECOG online database (https://precog.stanford.edu/, accessed on Oct. 12, 2023) showed the survival meta-Z score of HRH1 expression among 37 types of cancer. The Depmap portal online database (https://depmap.org/portal/; accessed on Apr. 29, 2022) presented the correlation between the HRH1 expression in lung cancer cell lines and the cytotoxicity of loratadine (17). The UALCAN online database (http://ualcan.path.uab.edu, accessed on Nov. 10, 2023) (18) was utilized to compute protein expression levels of PP2A-C, along with clinicopathologic parameters such as the clinical stage and tumor grade in LUAD, within the Clinical Proteomic Tumor Analysis Consortium (CPTAC) dataset. The Kaplan-Meier (KM) plotter database (https://kmplot.com/analysis/, accessed on Nov. 8, 2023) was employed to assess the impact of PP2A levels on the survival of LUAD subjects, utilizing data obtained from Gene Expression Omnibus (https://www.ncbi.nlm.nih.gov/geo/), including GSE102287, GSE14814, GSE19188, GSE29013, GSE30219, GSE31210, GSE31908, GSE37745, GSE43580, GSE50081, GSE77803, GSE8894, GSE68465 and GSE3141; and The Cancer Genome Atlas (https://www.cancer.gov/ccg/research/genome-sequencing/tcga), TCGA-LUAD, datasets. Patients were categorized into two groups based on high and low expression levels of PP2A-C, as determined by the best cutoff values for gene expression.

### Chemicals and materials

Loratadine (cat. no. 15625), desloratadine (cat. no. 16931), fexofenadine (cat. no. 18191), and the PP2A inhbitor, okadaic acid (OA; cat. no. 10011490) were purchased from Cayman Chemical Company. Histamine (cat. no. H7125), chloroquine (CQ; cat. no. C6628) and acridine orange (AO; cat. no. A9231) were obtained from MilliporeSigma. Antibodies of cleaved-PARP (cat. no. 5625), PARP (cat. no. 9532), cleaved-caspase-3 (cat. no. 9664), caspase-3 (cat. no. 9665), cleaved-caspase-8 (cat. no. 8582), cleaved-caspase-9 (cat. no. 7237), caspase-9 (cat. no. cs9508), p62 (cat. no. 5114), LC3 (cat. no. 4108), PP2A-C (cat. no. 2259), phosphorylated (p)-STAT3 (Tyr705; cat. no. 9145), STAT3 (cat. no. 9139), p-JNK (cat. no. 9251), p-extraceellular signal-regulated kinase (ERK; cat. no. 4370), ERK (cat. no. 4695), p-p38 (cat. no. 4511) and p38 (cat. no. 9212) were obtained from Cell Signaling Technology, Inc. HRH1 (cat. no. sc-374621), beclin (cat. no. sc-48341), JNK (cat. no. sc-7345) and GAPDH (cat. no. sc-32233) antibodies were obtained from Santa Cruz Biotechnology, Inc. An antibody specific for the phosphorylated form of PP2A-Cα (Tyr307) was purchased from Thermo Fisher Scientific, Inc. Lentiviral soups of small hairpin (sh)HRH1 were obtained from the RNA Technology Platfrom and Gene Manipulation Core at Academic Sinica. Targeting sequences of shRNAs were as follows: HRH1 shRNA-1: GCTCTGGTTCTATGCCAAGAT and HRH1 shRNA-2: CCTCTGCTGGATCCCTTATTT.

### Cell lines and cell culture

The study utilized five LUAD cell lines. A549, H23, H1975 and HCC827 were purchased from the American Type Culture Collection (ATCC). PC9 cells were obtained from the National Cancer Center Hospital (Tokyo, Japan). Cancer cell lines were cultured in RPMI-1640 medium with 10% fetal bovine serum (Gibco; Thermo Fisher Scientific, Inc.) and 1% penicillin-streptomycin-glutamine (Thermo Fisher Scientific, Inc.). Incubation for all cell types occurred at 37°C in a humidified incubator with 5% CO_2_. All cell lines were verified by short tandem repeat profiling analysis (Mission Biotech, Co., Ltd.). Cells were regularly checked for mycoplasma infection using EZ-PCR mycoplasma detection kit (Sartorius AG). All cell lines were performed with mycoplasma-free cells.

### Cell survival assay

In total, 3,000 HRH1-knockdown or control LUAD cells were seeded into single wells of a 96-well plate after treating cells with varying concentrations of loratadine or desloratadine for either 24 or 48 h. Cell viability was assessed using 100 *μ*l medium contain 10% Cell Counting Kit-8 (CCK-8; cat. no. 96992; MilliporeSigma) in a well. The assay was performed at 37°C, and absorbance (OD 450 nm) was measured every 1 h for up to 4 h. All measurements were performed using a CLARIOstar plate reader (BMG Labtech GmbH).

### Plate colony-forming assay

LUAD cells were initially seeded in six-well plates at a density of 10^3^ cells/well and allowed to incubate for 24 h. Following this, cells were treated with loratadine or desloratadine for an additional 24 h. Subsequently, the culture medium was refreshed every 2 days. After 7~14 days of incubation until a colony contained >50 cells, cells were fixed with 4% paraformaldehyde at room temperature for 20 min, and then stained with 0.1% crystal violet at room temperature for 30 min. Images of violet-stained colonies were captured and quantified by dissolving the violet stain in 10% acetic acid. To remove the stain, plates were placed on a shaker for 15 min, and the destaining solution was measured using a iMark™ microplate absorbance reader (Bio-Rad Laboratories, Inc.) at 595 nm.

### Western blot assay

Proteins were extracted using a PRO-PREP protein extraction solution (iNtRON Biotechnology, Inc.), and protein concentrations were determined as previously described (19). In total, 20 *μ*g of protein lysate was loaded into individual wells of 10% SDS-PAGE, and the entire gel was subsequently transferred onto a PVDF membrane (Bio-Rad Laboratories, Inc.). The blocking reagent (WesternF1; LionBIO, Inc.) was applied at room temperature for 1 min. The primary antibody was diluted at a 1:1,000 ratio in 1% BSA TBST buffer (0.1% Tween-20) and incubated overnight at 4°C. The anti-rabbit/mouse secondary antibody (cat. no. 12-348/12-349; MilliporeSigma) was diluted at a 1:2,000 ratio in TBST buffer and incubated for 1 h at room temperature. Separated proteins were then probed with respective antibodies and visualized using an enhanced chemiluminescence (ECL) system (Pierce Biotechnology; Thermo Fisher Scientific, Inc.). The intensity of band was measured by ImageJ version 1.54f (National Institutes of Health).

### Analysis of the sub-G_1_ phase

In total, 3.0×10^5^ LUAD cells were initially seeded in 6-cm dishes. Following overnight incubation, 10 and 30 *μ*M of loratadine were introduced. Cells were collected 24 h after treatment, fixed using 70% ice-cold ethanol for 5 min on ice, and then stained with a propidium iodide (PI) staining solution at room temperature for 1 h in the dark. Stained cells were left at room temperature in the dark for 0.5 h. Subsequently, flow cytometry (CytoFLEX; Beckman Coulter, Inc.) was employed to measure the cells, and their distribution into different intensities indicated the DNA content in distinct phases of the cell cycle. The sub-G_1_ phase was identified as the population with a DNA intensity lower than the threshold corresponding to the lowest DNA intensity in the G_0_/G_1_ peak. Each experiment was replicated three times. The analysis was proceeded by CytExpert 2.0 software (Beckman Coulter, Inc).

### Terminal deoxynucleotide transferase dUTP nick end labeling (TUNEL) stain

In total, 3×10^5^ LUAD cells were initially placed in 6-cm dishes. Following overnight incubation, 10 and 30 *μ*M of loratadine were introduced. Cells were collected at 24 h after treatment, fixed with 4% paraformaldehyde at room temperature for 20 min, and then treated with 70% ice-cold ethanol for 5 min on ice. Subsequently, they were digested with proteinase K (20 *μ*g/ml). Terminal ends of DNA fragments in dead cells were labeled with BrdU using the terminal deoxynucleotidyl transferase (TdT) enzyme. To detect signals, a fluorescein-labeled anti-BrdU antibody (Enzo Life Sciences, Inc.) was utilized, and measurements were taken using flow cytometry (CytoFLEX) and analyzed by CytExpert 2.0 software. Tumor tissue slides intended for TUNEL staining underwent dewaxing with Neo-Clear^®^ (MilliporeSigma) and subsequent rehydration with a series of ethanol solutions. The staining procedure then followed the same steps as those used for cell staining after ethanol fixation.

### Detection of acidic vesicular organelles (AVOs) via AO staining

Autophagy is the process of enclosing cytoplasmic proteins within a lytic compartment, marked by the formation and buildup of AVOs. To visualize these acidic cellular compartments, AO was employed, which emits bright-red fluorescence within acidic vesicles while displaying green fluorescence in the cytoplasm and nuclei. LUAD cells (3×10^5^ per well) were plated in 6-cm dishes and exposed to 10 or 30 *μ*M loratadine for 24 h. Following this, AO was introduced at a final concentration of 1 mg/ml for a 15-min incubation period at room temperature. Subsequently, images were captured using a fluorescence microscope (Zeiss AG).

### Lentiviral production and infection

The second generation lentivirus system was obtained from RNA Technology Platform and Gene Manipulation Core (Academia Sinica). Three lentiviral packaging vectors were introduced into 293T packaging cells (ATCC) through a calcium phosphate transfection method. To elaborate, 10^6^ 293T cells were transfected with 10 *μ*g of an HRH1 shRNA-expressing plasmid (pLKO-shHRH1-puro), in conjunction with 10 *μ*g of pCMVDR8.91 (the packaging vector) and 1 *μ*g of pMD.G (the envelope vector). Following a 16-h incubation at 37°C, transfection medium was replaced with fresh culture medium. After 48 h, the medium containing the lentivirus was collected from transfection, and cell debris was separated by centrifugation at 380 × g for 5 min at room temperature. The virus titer was measure by calculating the virus particle to generate infected colony by selecting with 5 *μ*g/ml puromycin. A multiplicity of infection of 5 was applied for further experiments. Subsequently, LUAD cells were exposed to the fresh lentivirus-containing medium, which was supplemented with 8 *μ*g/ml polybrene, for a 24-h period. The knockdown efficiency was assessed using a reverse-transcription quantitative polymerase chain reaction (RT-qPCR).

### RT-qPCR

Total RNA was isolated from LUAD cells with HRH1-knockdown using the TRIzol^®^ reagent (Thermo Fisher Scientific, Inc.). Subsequently, it was reverse-transcribed into complementary (c)DNA using the iScript™ cDNA Synthesis kit (Bio-Rad Laboratories, Inc.) according to the mnufacturer's protocol. The generated cDNA was then utilized in an RT-qPCR, by applying the SYBR qPCR supermix reagent (Bio-Rad Laboratories, Inc.). The thermocycling condition was an initial denaturation at 95°C for 10 min and a 40 cycles of two steps PCR at 95°C for 15 sec and 60°C for 30 sec, then a dissociation stage for melting curve analysis. Results were recorded with a 7300 Real-Time PCR system (Applied Biosystems; Thermo Fisher Scientific, Inc.) and the 2^−ΔΔCq^ method was used for quantification (20). Primers used in the RT-qPCR are listed as follows: HRH1 forward, 5′-GCC GAGAGGACAAGTGTGA-3′ and reverse, 5′-GGAGACTCCTTCCCTGGTTT-3′; and GAPDH forward, 5′-CTGGAGAAACCTGCCAAGTATGAT-3′; and reverse, 5′-TTCTTACTCCTTGGAGGCCATGTA-3′.

### In vivo xenograft model

In this xenograft tumor model, a total of 14 8-week-old non-obese diabetic (NOD)-SCID male mice were employed. The body weight at the beginning was 20-25 g. Mice were housed in an individually ventilated cage (IVC) system under controlled conditions: Temperature maintained at 20°C, total air exchange, relative humidity at 55±5%, a 12/12-h light/dark cycle, and with *ad libitum* access to sterilized water and food. A total of 5 million PC9 cells were subcutaneously injected into the back of each mouse in a 100-*μ*l phosphate-buffered saline suspension. Following cell transplantation, loratadine [10 mg/kg body weight (BW)] was administered orally by gavage on a daily basis. There were seven mice in each group. Treatment was administered on weekdays, with weekends serving as rest days, until the conclusion of the experiment. The tumor size, measured in cubic millimeters with the formula 1/2 ab^2^ (where 'a' represents length and 'b' represents width), was recorded weekly. Additionally, BWs of the mice were recorded on a weekly basis. Each group consisted of seven mice. At the end of the experiment, the mice were humanely euthanized by CO_2_ asphyxiation within their home cages. A controlled CO_2_ flow rate of 3 l/min was maintained for at least 5 min to ensure complete and irreversible loss of consciousness. This resulted in an air displacement rate of ~40% of the chamber volume per min. Primary tumor specimens were resected, images were captured, fixed, and sectioned for hematoxylin and eosin (H&E) and immunohistochemical (IHC) staining. All animal experiments were conducted in accordance (approval no. wan-lac-110-026) with guidelines of the Institutional Animal Care and Use Committee (IACUC) of Wan Fang Hospital (Taipei, Taiwan).

### IHC of tumor specimens

All tumor tissue samples were fixed in a 10% buffered formaldehyde solution at room temperature overnight, embedded in paraffin blocks, and cut into 5-*μ*m sections. Paraffin-embedded LUAD tissue sections were deparaffinized using Neo-Clear^®^ (MilliporeSigma) and rehydrated through a gradient of ethanol concentrations. Subsequently, slides underwent high-pressure incubation in antigen retrieval buffer (pH 6.0; DAKO; Agilent Technologies, Inc.) for 30 min, followed by blocking with super block buffer (ScyTek Laboratories, Inc.) for 1 h, then overnight incubation at 4°C with the anti-Ki-67 (cat. no. 9027S; Cell Signaling Technology, Inc.), anti-CD31 (cat. no. 77699S; Cell Signaling Technology, Inc.), or anti-LC3 antibody. Primary antibodies were diluted with with antibody dilution buffer (cat. no. ADB250; Ventana; Roche Tissue Diagnostics) at 1:100 ratio. Next, a HRP rabbit/mouse reagent was applied and incubated with the slides at room temperature for 1 h, with the Dako REAL EnVision system (cat. no. K5007; DAKO; Agilent Technologies, Inc.) being used to visualize the binding of the antibody to the tissue. Hematoxylin was used as a counterstain. Finally, the cover slides were sealed, and slides were scanned using the light motic digital pathology system, MoticEasyScan Pro 6.

### Statistical analysis

The *in vitro* and *in vivo* study results are presented as the mean ± standard deviation (SD). Pearson's correlation coefficients were used to assess the relationship between gene expression and drug sensitivity. Survival analysis was conducted using the Kaplan-Meier method combined with the log-rank test. The Z-value of PPP2CA indicates the SD from the median across lung cancer samples, while log2 spectral count ratio values from CPTAC were normalized within individual sample profiles and subsequently across all samples. Comparisons involving more than three groups and varying drug concentrations were analyzed using ANOVA with Bonferroni post hoc tests. Comparisons between two groups were performed using an unpaired, two-tailed Student's t-test, with statistical significance threshold set at a P<0.05.

## Results

### HRH1 expression is correlated with poor prognoses in patients with LUAD

**Figure 1:**
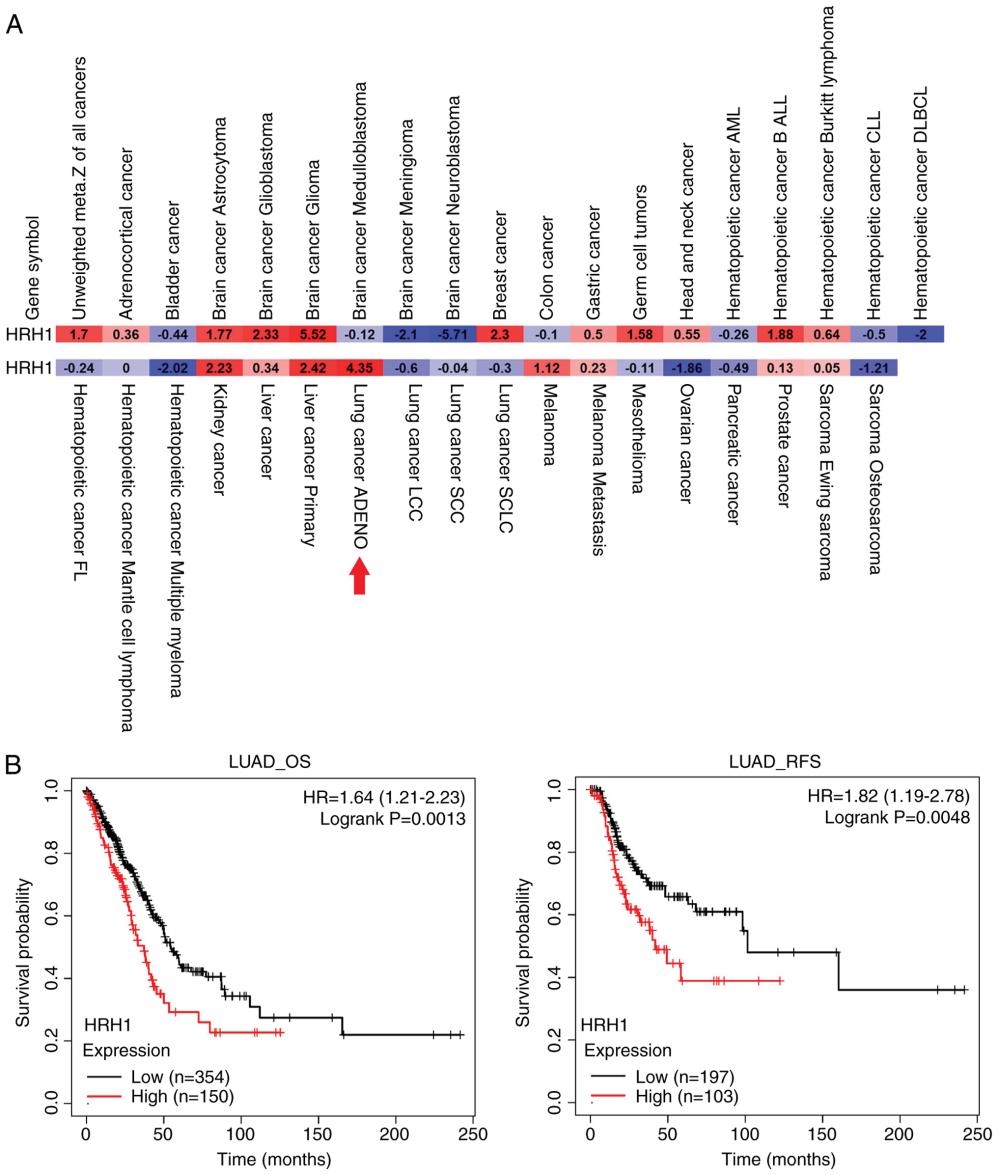
Prognostic significance of HRH1 in LUAD. (A) The pan-cancer expression of HRH1 levels by a meta-Z analysis obtained from the PRECOG website. (B) Associations between HRH1 expression and OS (left panel) and RFS (right panel) in patients with LUAD using the Kaplan-Meier plotter database. Gene expression levels are stratified into high and low using the best cutoff value, with statistical significance defined as P<0.05. HRH1, histamine receptor H1; LUAD, lung adenocarcinoma; OS, overall survival; RFS, relapse-free survival; HR, hazard ratio.

Because the use of HRH1 antagonists was reported to reduce the risk of liver cancer development (21) and improve survival rates in patients with melanoma and breast cancer (8,9), an analysis of HRH1 expression levels and their prognostic significance was initiated in 39 different cancer types using the pan-cancer prognostic database, PRECOG. Results indicated that HRH1 exhibited prognostic potential for unfavorable outcomes in various human cancers, including LUAD (Fig. 1A). Furthermore, an analysis of associations of HRH1 expression with overall survival (OS) and relapse-free survival (RFS) was conducted in patients with LUAD using the KM plotter (Fig. 1B). It was found that higher levels of HRH1 were associated with poorer OS and RFS in patients with LUAD. Collectively, these clinical data suggest that HRH1 expression may promote the progression of LUAD, indicating that HRH1 antagonists hold promise as a potential treatment for LUAD.

### HRH1 antagonists induce proliferation inhibition and apoptotic cell death in human LUAD cells harboring different EGFR statuses

**Figure 2:**
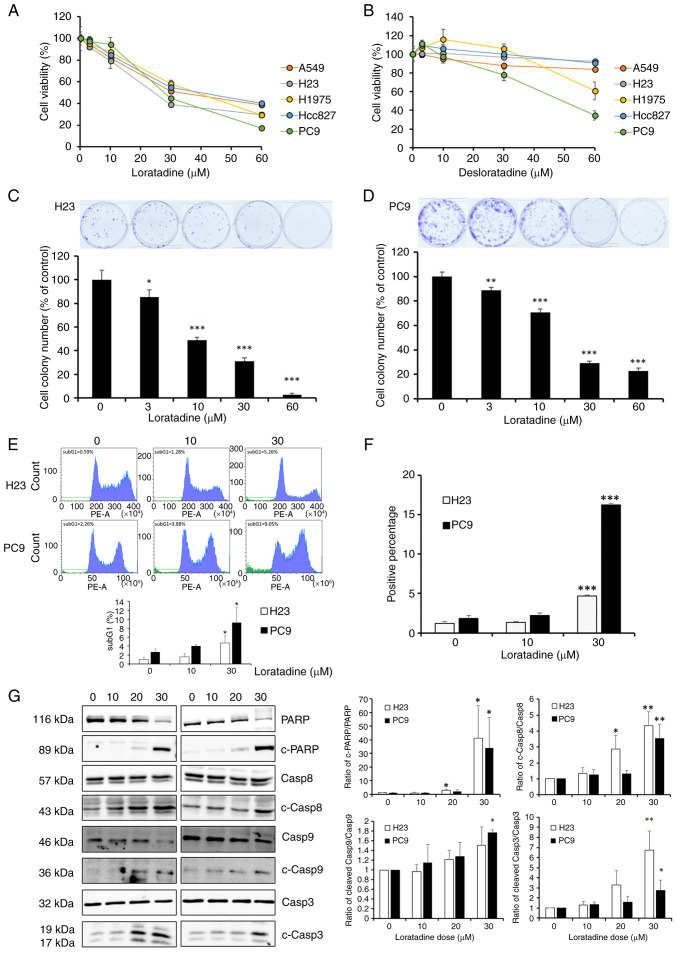
Loratadine induces apoptotic cell death of LUAD cells with wild-type or mutant EGFR. (A and B) LUAD cells with various EGFR statuses were exposed to different concentrations of (A) loratadine and (B) desloratadine for 48 h, followed by an assessment of cell viability using a Cell Counting Kit-8 assay. (C and D) H23 and PC9 cells were treated with either the vehicle or loratadine (3-60 *μ*M) for 24 h, and the long-term growth inhibitory effects of loratadine on cell colonies were evaluated. Upper panels of C and D: representative photomicrographs. Values in A-D are expressed as the percentage of cell inhibition, with vehicle-treated cells considered 100%. (E-G) H23 and PC9 cells were exposed to 10 and 30 *μ*M loratadine for 24, stained with (E) PI or (F) TUNEL, and then analyzed by flow cytometry. Percentages of cells in the sub-G_1_ phase and positive TUNEL staining were determined. (G) H23 and PC9 cells were treated with various concentrations of loratadine for 24 h, and levels of apoptosis-associated proteins were assessed through a western blot analysis. GAPDH was used as a loading control. Data from E to G are presented as the mean ± SD. Data from panels C to G were analyzed using one-way ANOVA. ^*^P<0.05, ^**^P<0.01 and ^***^P<0.001 vs. the control group. LUAD, lung adenocarcinoma; EGFR, epidermal growth factor receptor.

To further explore the extensive therapeutic potential of HRH1 antagonist loratadine in the context of LUAD, an investigation was conducted into the impacts of various concentrations of loratadine and its metabolite, desloratadine, on the viability of LUAD cells with distinct EGFR statuses. These statuses included WT EGFR in A549 and H23 cells, EGFR exon 19 deletion in PC9 and HCC827 cells, and L858R/T790M mutations in H1975 cells. The present results revealed that both loratadine and desloratadine exhibited cytotoxic effects on all tested LUAD cell lines in the concentration range of 30~60 *μ*M, as assessed by a CCK-8 viability assay. Notably, loratadine exhibited a more-potent inhibitory effect on the viability of LUAD cells compared with desloratadine (Fig. 2A and B). As a result, subsequent experiments were conducted using a specific concentration range of loratadine. Unlike the toxic effects observed on LUAD cells, loratadine treatment at the same concentrations resulted in no significant toxicity or only minor effects on BEAS-2B normal lung epithelial cells (data not shown). The effect of loratadine on the long-term growth of LUAD cells was further assessed through a colony-formation assay. This assay revealed a concentration-dependent reduction in the number of cancer cell colonies following loratadine treatment (Fig. 2C and D). These findings suggested that loratadine holds promise as a therapeutic agent for managing LUAD, in cases of both WT and mutant EGFR. To investigate the mode of the antiproliferative effects induced by loratadine, H23 and PC9 cells were treated with 10 and 30 *μ*M loratadine for 24 and 48 h. A flow cytometry-based cell cycle analysis revealed increased accumulation of cells in the sub-G_1_ phase after 24 h of treatment with 30 *μ*M loratadine (Fig. 2E). Furthermore, DNA fragmentation was evaluated using TUNEL staining and increased percentages of TUNEL-positive cells were observed in H23 and PC9 cells treated with 30 *μ*M loratadine compared with control cells (Fig. 2F). Additionally, expression levels of apoptotic markers, including cleaved caspase-8/-9/-3 and PARP, were concentration-dependently induced after 24 h of treatment with different loratadine concentrations (10~30 *μ*M) (Fig. 2G). Results collectively represented typical characteristics of apoptotic cell death and underscored loratadine's ability to induce apoptosis in LUAD cells.

### The antiproliferative ability of loratadine against LUAD cells is independent of HRH1

**Figure 3:**
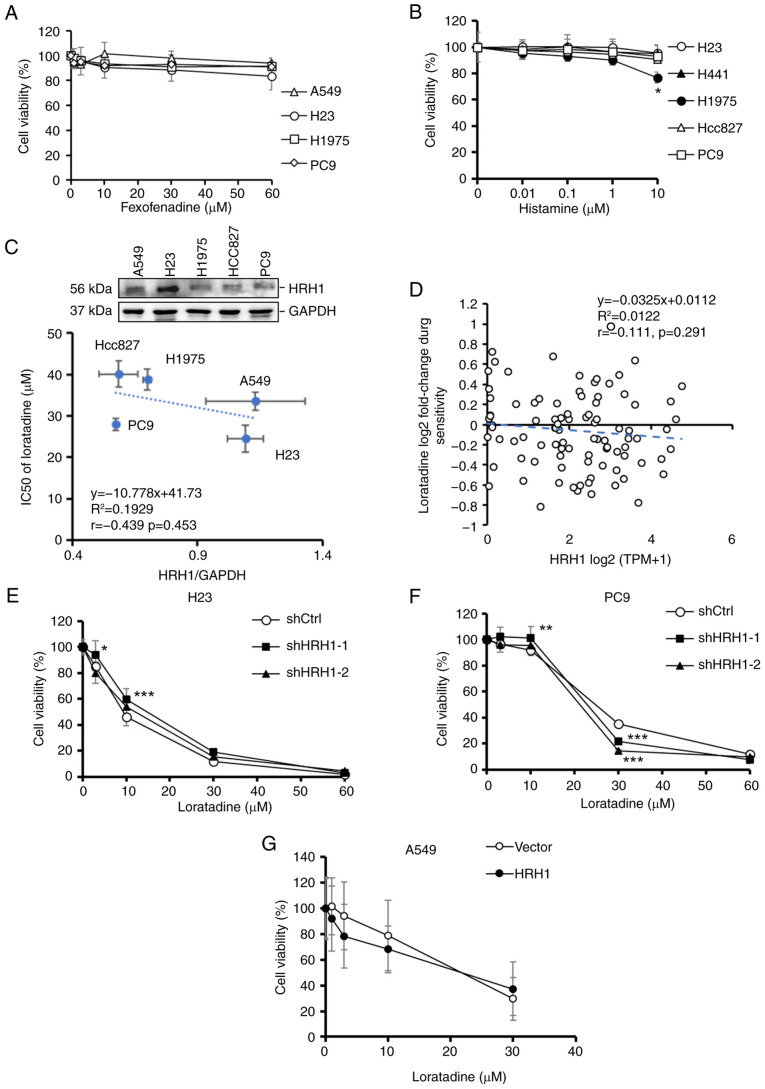
Antiproliferative ability of loratadine on LUAD cells is independent of HRH1. (A and B) LUAD cells with different epidermal growth factor receptor statuses were exposed to varying concentrations of (A) fexofenadine and (B) histamine for 24 h, and their cell viability was assessed with a Cell Counting Kit-8 assay. Values are expressed as a percentage of vehicle-treated cells, with vehicle-treated cells set as 100%. Data are presented as the mean ± SD. (C) Upper panel: Endogenous HRH1 levels in various LUAD cell lines were determined via a western blot analysis. Lower panel: Correlations between HRH1 protein expression levels and the IC_50_ of loratadine in LUAD cell lines. (D) Correlations between HRH1 mRNA expression and the IC_50_ of loratadine using data from lung cancer cell lines in the DepMap database. (E-G) Cell viability of (E) H23, (F) PC9 and (G) A549 cells, which were respectively subjected to HRH1 knockdown and overexpression, followed by loratadine treatment at various concentrations for an additional 24 h. Values are expressed as a percentage of vehicle-treated cells, with vehicle-treated cells set as 100%. Data are presented as the mean ± SD. Data from panels A, B, and E-G were analyzed using two-way ANOVA. ^*^P<0.05, ^**^P<0.01 and ^***^P<0.001 vs. the control group. LUAD, lung adenocarcinoma; HRH1, histamine receptor H1; IC_50_, 50% inhibitory concentration; sh-, short hairpin.

In addition to loratadine, the effects of another commonly used HRH1 antagonist, fexofenadine, were tested on LUAD proliferation. Surprisingly, it was observed that fexofenadine, despite having a different chemical structure than loratadine, did not exhibit a significant cytotoxic effect on various LUAD cell lines, including A549, H23, H1975 and PC9 cells (Fig. 3A). Moreover, treatment of various LUAD cell lines with the HRH1 ligand histamine at different concentrations showed no significant impact on cell proliferation rates, except for 10 *μ*M histamine in H1975 cells. (Fig. 3B). Furthermore, it was observed that there was no significant correlation (*r*=-0.439, P=0.453) between the HRH1 protein expression level and 50% inhibitory concentration (IC_50_) values of loratadine in the LUAD cells tested (Fig. 3C). To further validate the aforementioned results, a correlation analysis was performed using DepMap to investigate the relationship between HRH1 RNA expression in lung cancer cell lines and the cytotoxicity of loratadine (Fig. 3D). The analysis revealed no significant correlation between HRH1 expression and loratadine sensitivity (Pearson correlation *r*=-0.111, P=0.291). These results suggested that loratadine-induced cytotoxicity was independent of HRH1. To further investigate whether the antiproliferative effect of loratadine was mediated via HRH1, stable shHRH1 clones were established in various LUAD cell lines with different EGFR statuses, including H23 and PC9 cells (Fig. S1A and B). Regardless of whether cells expressed the control vector or HRH1 shRNAs, a similar trend of proliferation inhibition was observed upon loratadine treatment. However, certain concentrations showed significant differences compared with the control, such as 3 and 10 *μ*M in H23-shHRH1-1 cells, 10 and 30 *μ*M in PC9-shHRH1-1 cells, and 30 *μ*M in PC9-shHRH1-2 cells (Fig. 3E and F). In contrast to HRH1-knockdown, overexpressing HRH1 in A549 cells (Fig. S1C) did not significantly affect the cytotoxic effect of loratadine (Fig. 3G). Taken together, these results suggested that HRH1 does not appear to be the major factor involved in loratadine-induced cell death.

### Loratadine induces autophagy-mediated apoptotic cell death in LUAD cells

**Figure 4:**
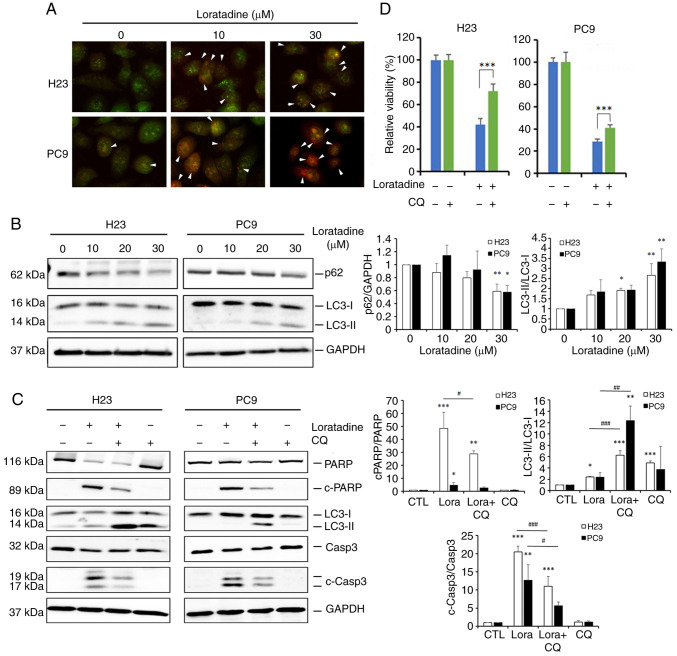
Loratadine induces autophagy-mediated apoptotic cell death in lung adenocarcinoma cells. (A) H23 and PC9 cells were treated with loratadine (10 and 30 *μ*M) for 24 h. Lysosomal membrane stability was measured by acridine orange staining under a fluorescence microscope. Autophagy was indicated by cells exhibiting bright-red fluorescence (white arrowheads). The image was captured at a magnification of ×40. (B) H23 and PC9 cells were treated with loratadine at the indicated concentrations for 24 h. LC3 conversion (LC3-I to LC3-II) and expression of p62 was detected by western blot analysis. GAPDH was used as a loading control. (C and D) H23 and PC9 cells were pretreated with CQ (20 *μ*M) for 1 h, followed by loratadine (30 *μ*M) treatment for 24 h. LC3 conversion, c-PARP and c-caspase-3 levels, and cell viability in both cell lines were detected by western blot analysis and Cell Counting Kit-8 assay, respectively. Values, expressed as the percentage of cell inhibition, considered vehicle- or CQ-treated cells as 100%. Data are presented as the mean ± SD. Data from panels B and C were analyzed using one-way ANOVA. ^*^P<0.05, ^**^P<0.01 and ^***^P<0.001 vs. the control group. ^#^P<0.05, ^##^P<0.01 and ^###^P<0.001 vs. the loratadine-treated group. LC3, light chain 3; CQ, chloroquine; PARP, poly (ADP ribose) polymerase; c-, cleaved.

Autophagy, a form of type-II programmed cell death, is currently a highly studied field in cancer biology. Terfenadine, an HRH1 antagonist, was reported to induce both apoptosis and autophagy in melanoma cells (13). Since terfenadine and loratadine both belong to the class of cationic amphiphilic antihistamines (22), it was investigated whether autophagy plays a role in the cell death induced by loratadine in LUAD cells. Initially, the potential for autophagy induction by loratadine was assessed through detecting AVOs using AO staining. It was found that loratadine-treated H23 and PC9 cells displayed AVOs, which were identified as red fluorescent spots. The number of AVOs was higher in loratadine-treated cells than in untreated cells (Fig. 4A). Furthermore, the conversion of LC3-I to LC3-II and levels of beclin 1 and p62, an autophagosome component or target, was examined using western blotting. Indeed, loratadine treatment of H23 and PC9 cells caused the LC3-I to -II conversion and p62 downregulation in a concentration-dependent manner (Fig. 4B). Additionally, loratadine treatment led to an increase in beclin 1 expression (data not shown). These results collectively indicated that loratadine treatment induces autophagy in LUAD cells. Next, to explore the interplay between autophagy and apoptosis induced by loratadine, the autophagy inhibitor, CQ, was employed. Pretreatment of H23 and PC9 cells with CQ significantly reversed the cleavage of caspase-3 and PARP induced by loratadine, compared with loratadine treatment alone (Fig. 4C). Moreover, changes in H23 and PC9 cell death was examined following treatment with loratadine, both with and without CQ. CCK-8 assays revealed that CQ significantly mitigated loratadine-induced cell death (Fig. 4D), suggesting that autophagy induction enhanced the apoptotic effect initiated by loratadine in LUAD cells.

### Loratadine exhibits a significant antitumor effect in the PC9 xenograft model by suppressing angiogenesis and promoting autophagy and apoptosis

**Figure 5:**
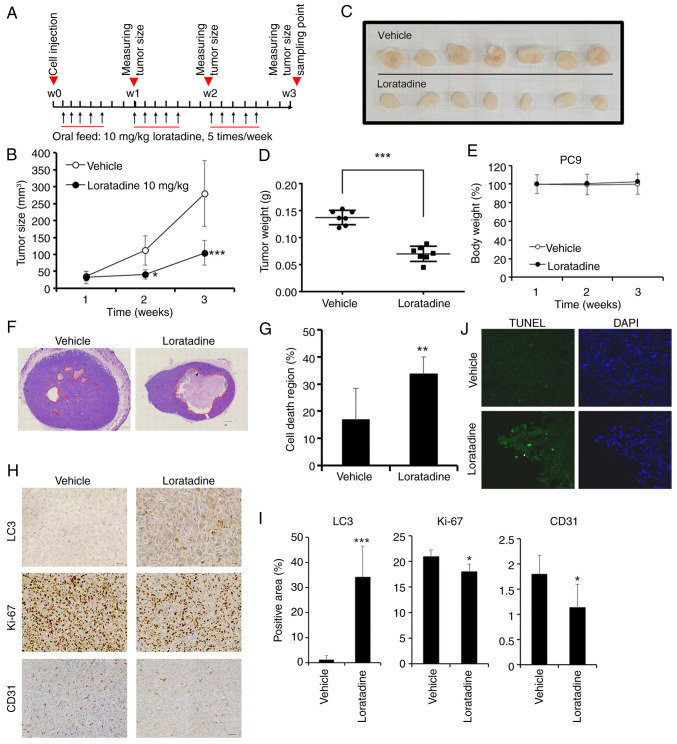
Anticancer growth effects of loratadine in a PC9 xenograft model. (A) Timeline of the *in vivo* study design for investigating anticancer activities of loratadine. PC9 cells were subcutaneously injected into the back of NOD-SCID mice which were subsequently orally fed loratadine (10 mg/kg body weight), five times per week for a duration of 21 days (B) Average tumor volume of vehicle-treated (open circles, n=7) versus loratadine-treated (filled circles, n=7) NOD-SCID mice. Data were analyzed by two-way ANOVA. (C) Gross appearance of subcutaneous tumors after treatment with vehicle or loratadine for 21 days. (D) Tumor weights were compared between loratadine-treated (n=7) and vehicle-treated (n=7) tumor-bearing mice at the end of the study. (E) Body weight of each mice was measured weekly. Values were normalized by using the average value of first week as 100%. (F) Cross-section of tumor tissues from animals treated with loratadine or vehicle were stained with H&E. Scale bar, 600 *μ*m. (G) Measurement of the area of the cell death region in cross-sections of tumor tissues from both vehicle- and loratadine-treated groups. (H) PC9 tumor tissue sections from animals treated with vehicle or loratadine were IHC-stained for LC3, Ki-67, or CD31, and counterstained with hematoxylin. Scale bar, 60 *μ*m. (I) Quantitative analysis of LC3, Ki-67, and CD31 IHC staining intensities using ImageJ software. (J) Fluorescent TUNEL-stained death part of tumor tissues with loratadine treatment compared with the vehicle. DAPI stain presented the cell distribution and the morphology of nucleus. Values represent the mean ± SD. ^*^P<0.05, ^**^P<0.01 and ^***^P<0.001 compared with the vehicle-treated group. IHC, immunohistochemical; LC3, light chain 3; CD31, cluster of differentiation 31.

To assess the *in vivo* inhibitory properties of loratadine, xenograft mouse models were created using PC9 cells. Loratadine was administered through oral gavage five times a week, as illustrated in Fig. 5A. On day 21, the average tumor volume in mice treated with loratadine was smaller compared with mice received the vehicle, as depicted in Fig. 5B. Moreover, tumor weights of xenografts removed from the loratadine-treated group were significantly lower than those from the control group (Fig. 5C and D). The average weight of tumors treated with loratadine was reduced by ~56% compared with tumors treated with the vehicle (Fig. 5D). Furthermore, the treatment dosage of loratadine (10 mg/kg BW) did not affect BWs of mice (Fig. 5E). A histopathological analysis of tumor tissues by H&E staining unveiled a notable region of cell death in the loratadine-treated group, encompassing ~33.6±6.2% of the cross-sectional area (P=0.002), in contrast to the vehicle-treated group, where it was only 16±11% (Fig. 5F and G). In support of the anticancer mechanism in loratadine-treated LUAD cells, proliferative, autophagic, angiogenic and apoptotic signals within tumor tissues were assessed through IHC staining for Ki67, LC3 and cluster of differentiation 31 (CD31), as well as immunofluorescent staining for BrdU. It was revealed that the proliferation marker, Ki67, and the angiogenic marker, CD31, exhibited reduced expression in the loratadine-treated group compared with the vehicle-treated group. Conversely, the autophagy marker, LC3, displayed a significant increase in tumor tissues treated with loratadine (Fig. 5H and I). Additionally, TUNEL staining demonstrated an increase in apoptotic cells in the loratadine-treated group compared with the vehicle-treated group (Fig. 5J). In summary, the anticancer efficacy of loratadine in the PC9 xenograft model is attributed to its ability to inhibit proliferation and angiogenesis, as well as to induce autophagy and apoptosis.

### Loratadine triggers autophagy-mediated apoptotic cell death via inducing the deactivation of the p38, JNK and STAT3 pathways

**Figure 6:**
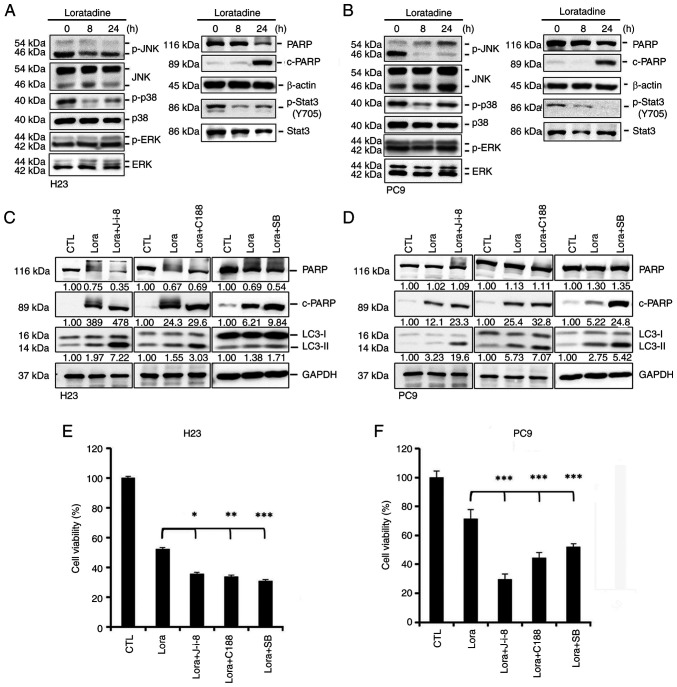
Loratadine induces autophagy-mediated apoptotic cell death via deactivating p38, c-JNK and 3 STAT3 pathways in lung adenocarcinoma cells. (A and B) Phosphorylation levels of JNK1/2, p38, ERK1/2 and STAT3 and the expression level of c-PARP were assessed using western blot analysis after treating (A) H23 or (B) PC9 cells with 30 *μ*M loratadine for the indicated time points. (C and D) H23 and PC9 cells were pretreated with and without 5 *μ*M JNK-in-8, 30 *μ*M C188, or 10 *μ*M SB203580 for 1 h followed by 30 *μ*M loratadine treatment for an additional 24 h. Expression levels of c-PARP and LC3 conversion were determined by western blot analysis. GAPDH was used as a loading control. (E and F) H23 and PC9 cells were treated as aforementioned and cell viability changes of both cells were determined by a Cell Counting Kit-8 assay. Values are expressed as the percentage of cell inhibition, with vehicle-treated cells considered 100%. Data are presented as the mean ± SD. Data were analyzed by one-way ANOVA. ^*^P<0.05, ^**^P<0.01 and ^***^P<0.001 compared with the loratadine-treated group. c-, cleaved; JNK, Jun N-terminal kinase; STAT3, signal transducer and activator of transcription 3; ERK1/2, extracellular signal-regulated kinase 1/2; PARP, poly (ADP ribose) polymerase; LC3, light chain 3; p-, phosphorylated.

Previous research indicated the association of the mitogen-activated protein kinase (MAPK) and Akt signaling pathways with autophagy and apoptosis in cancer (23-25). In addition, the anticancer mechanisms of cationic amphiphilic antihistamines were linked to STAT3 inhibition (26). Consequently, it was investigated whether the MAPK, Akt and STAT3 pathways play roles in loratadine-induced autophagy and apoptosis in LUAD cells. Loratadine treatment for 8 and 24 h resulted in the inhibition of MAPK signals, specifically p38 and JNK, but not ERK, in H23 (Fig. 6A) and PC9 (Fig. 6B) cells. Furthermore, loratadine was found to inhibit STAT3 activation (Y705) in both cell lines (Fig. 6A and B). By contrast, loratadine treatment had no inhibitory effect on Akt phosphorylation in either LUAD cell line (Fig. S2). To examine whether the p38, JNK and STAT3 pathways were involved in loratadine-induced autophagy and apoptosis in LUAD cells, H23 and PC9 cells were pretreated with inhibitors: 10 *μ*M SB203580 (a p38 inhibitor), 5 *μ*M JNK-in-8 (a JNK inhibitor), or 30 *μ*M C188 (a STAT3 inhibitor) for 1 h. Subsequently, cells were treated with 30 *μ*M loratadine for another 24 h and analyzed using western blotting. The cleavage of PARP and LC3 turnover induced by loratadine were markedly amplified by the inhibitors SB203580, JNK-in-8 and C188 (Fig. 6C and D). Functionally, inhibition of the p38, JNK and STAT3 pathways further enhanced loratadine's suppressive effects on the viability of H23 and PC9 cells (Fig. 6E and F). Additionally, treatment with these inhibitors alone partially reduced the viability of both LUAD cell lines (Fig. S3). These findings collectively highlight the critical roles of the p38, JNK and STAT3 pathways in facilitating loratadine-induced, autophagy-mediated apoptosis in LUAD cells.

### The deactivation of p38 and JNK signals mediated by loratadine is dependent on PP2A activation, whereas the deactivation of STAT3 is independent of PP2A

**Figure 7:**
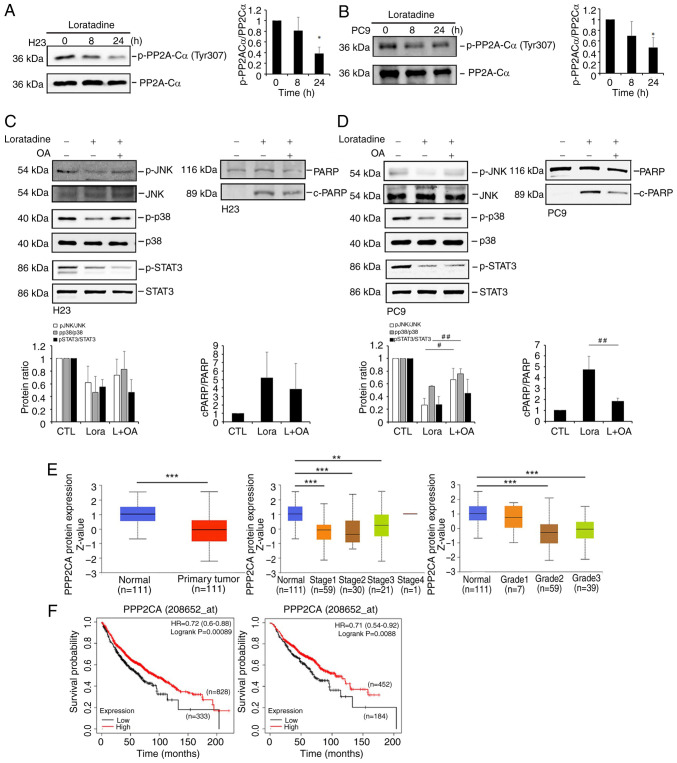
Loratadine-induced PP2A activation is responsible for the deactivation of p38 and c-JNK, but not for the STAT3 pathways in LUAD cells. (A and B) Phosphorylation level of PP2A-Cα at Tyr307 was assessed using western blot analysis after treating (A) H23 or (B) PC9 cells with 30 *μ*M loratadine for 8 and 24 h. (C and D) Pretreatment of H23 and PC9 cells with 5 nM OA for 1 h followed by 30 *μ*M loratadine treatment for an additional 24 h. Phosphorylation levels of p38, JNK, STAT3 and c-PARP were determined by western blot analysis. (E) UALCAN portal analysis of LUAD samples from the CPTAC dataset. Comparison of PP2A-C (PPP2CA) protein expression levels between normal and tumor tissues (left panel). Expression levels of PP2A-C protein levels in tumor tissues obtained from patients with LUAD at different clinical stages (middle panel) and tumor grades (right panel). ^**^P<0.01 and ^***^P<0.001 compared with the normal group. (F) Association between PPP2CA expression with overall survival in patients with LUAD (left panel) or a sub-population with negative surgical margins (right panel) as determined using a Kaplan-Meier plotter database. Gene expression levels were dichotomized into high and low values using the best cutoff value. P<0.05 was considered to indicate a statistically significant difference. Data are presented as the mean ± SD. Data from panels A-D were analyzed using one-way ANOVA. ^*^P<0.05 compared with the vehicle-treated group. ^#^P<0.05 and ^##^P<0.01 vs. the loratadine-treated group. PP2A, protein phosphatase 2A; c-, cleaved; JNK, Jun N-terminal kinase; STAT3, signal transducer and activator of transcription 3; LUAD, lung adenocarcinoma; OA, okadaic acid; PARP, poly (ADP ribose) polymerase; HR, hazard ratio; p-, phosphorylated.

It is worth noting that PP2A, a serine/threonine phosphatase, was shown to deactivate MAPKs (27) and STAT3 (28). Hence, it was hypothesized that loratadine might activate PP2A to deactivate MAPKs and STAT3, ultimately leading to suppression of LUAD growth. Indeed, treatment of H23 and PC9 cells with loratadine was observed to reduce the phosphorylation of PP2A at Tyr307 (Fig. 7A and B), a modification known to decrease its activity (29). Moreover, it was observed that pretreatment with the PP2A inhibitor, OA, was able to reverse the loratadine-induced deactivation of p38 and JNK, and cleavage of PARP in both H23 and PC9 cells (Fig. 7C and D). By contrast, the inhibition of p-STAT3 caused by loratadine was not reversed by OA pretreatment (7C and D). These findings suggested that the apoptotic effect induced by loratadine in LUAD cells may occur through activation of PP2A, which in turn negatively regulates p38 and JNK activities. In clinical settings, a significant reduction in PP2A protein levels was evident in LUAD tissues compared with normal tissues, as indicated by proteomic data sourced from the UALCAN database (18). Furthermore, a subgroup analysis was conducted considering various clinical and pathological factors related to LUAD and it was found that PP2A protein levels exhibited a decrease in LUAD clinical stages 1 to 3 and tumor grades 2 to 3 compared with normal samples (Fig. 7E). In addition, associations between PP2A and the OS of patients with LUAD were analyzed using the KM-plotter. As depicted in the left panel of Fig. 7F, patients with LUAD with high PP2A expression exhibited an extended OS compared with those with low PP2A expression. Moreover, high PP2A in patients with LUAD with negative surgical margins also had longer OS times compared with those with low PP2A expression (Fig. 7F, right panel).

## Discussion

A nationwide pharmacoepidemiological cohort study showed that the use of loratadine, a cationic amphiphilic H1 antihistamine, was correlated with significantly reduced all-cause mortality among patients with non-localized NSCLC (22), suggesting that loratadine may exhibit therapeutic potential for NSCLC. In the present study, it was found that loratadine inhibited the proliferation of LUAD cells harboring either WT or mutant EGFR in both *in vitro* and *in vivo* experiments. Conversely, fexofenadine, a non-cationic amphiphilic H1 antihistamine, did not exhibit a significant anticancer growth effect in LUAD cells. This result may echo previous clinical observations which indicated that loratadine use was associated with significantly reduced mortality compared with the use of the fexofenadine in patients with NSCLC (22). Previously, histamine was demonstrated to play an important role in modulating the proliferation of various cancer cells and was shown to act on HRH1 (30,31). However, the results of the present study demonstrated that histamine treatment did not affect the proliferation of various LUAD cells. Furthermore, both knockdown and overexpression of HRH1 had slight impacts on the cytotoxic effect of loratadine against LUAD cells. Collectively, these findings suggest that loratadine-induced cytotoxicity in LUAD cells is independent of HRH1. Similar to the current findings, another cationic amphiphilic H1 antihistamine, terfenadine, was shown to induce cell death independently of HRH1 in melanoma cells (32). Moreover, there is a recent study presenting the HRH1-independent anti-inflammatory effects of HRH1 antagonists by targeting a member of MAPK kinase kinase (MAP3K), TAK1, and suppressing consequent AP-1 signaling pathway activation (15).

Mechanistically, the present study demonstrated that autophagy and apoptosis were both induced in H23 and PC9 cells. The interplay between apoptosis and autophagy in loratadine-induced cytotoxicity was also emphasized. Autophagy was reported to act as either a guardian or executor of apoptosis in NSCLC, depending on the surrounding microenvironment, therapeutic interventions and stage of the carcinoma (33). The current research revealed that inhibition of autophagy by an autophagy inhibitor reduced loratadine-induced apoptotic cell death in LUAD cells, indicating that loratadine-induced autophagy serves as a pro-death mechanism rather than a prosurvival one. The role of autophagy in promoting or inhibiting apoptosis varies depending on specific molecular factors, numerous of which remain poorly understood. Mutations in the p53 tumor suppressor gene are among the most common genetic alterations in LUAD, occurring in 45-70% of cases (33). The nuclear transcriptional activity of WT p53 activates multiple target genes that promote both autophagy and apoptosis (33). Conversely, deletion, depletion, mutations, or pharmacological inhibition of p53 induces autophagy in human cells, protecting them from apoptosis under hypoxic or nutrient-starvation conditions, thereby suggesting an anti-apoptotic function for autophagy (34). Notably, a recent study revealed that loratadine treatment enhances p53 expression in a Lewis lung carcinoma xenograft model (35). Additionally, high level p53 activation was shown in H23 and PC9 LUAD cells (36) which were used in the present study. These findings suggest that patients with LUAD with WT p53 may be better suited for loratadine treatment compared with those with mutant p53. Various factors function in both apoptosis and autophagy. For instance, the interaction between caspases and autophagy-related (ATG) proteins in regulating apoptosis was previously documented. ATG4D can be cleaved by caspase-3 and recruited to mitochondria to stimulate apoptosis (37). Caspase-9 was shown to induce autophagy by increasing LC3 lipidation through interaction with ATG7 (38). While loratadine was demonstrated to activate caspase-9 and -3 in LUAD cells, further investigation is needed in the future to elucidate regulation of the crosstalk between caspases and ATGs by loratadine. Additionally, the Farnesoid X receptor (FXR)-oxidative stress-induced growth inhibitor 1 (OSGIN1) axis has been reported to promote autophagy, influencing various inflammatory-related disorders such as pancreatitis (39) and chronic obstructive pulmonary disease (40). Interestingly, both upregulation and downregulation of OSGIN1 were shown to enhance autophagy responses triggered by tobacco smoking in the human airway epithelium (41). In NSCLC, elevated levels of FXR and OSGIN1 were found to contribute to disease progression by activating STAT3 signaling (42) and modulating microtubule dynamics (43), respectively. However, it remains to be determined whether loratadine-induced autophagic responses play a role in the FXR-OSGIN1 axis-mediated progression of NSCLC.

Extensive research has highlighted the pivotal role of MAPKs in converting extracellular stimuli into a wide range of cellular responses, including cell growth, proliferation, apoptosis and autophagy. Among MAPKs, JNK and p38 MAPK were shown to mediate certain antiapoptotic processes (44). For example, JNK activation was reported to mitigate endoplasmic reticulum stress-induced cell death by enhancing expression levels of various antiapoptotic proteins, including cellular inhibitor of apoptosis protein 1 (cIAP1), cIAP2, X-linked inhibitor of apoptosis protein and baculoviral AIP repeat-containing 6 (45). Transient JNK activation was demonstrated to delay caspase-9 activation by directly interacting with apoptotic protease-activating factor-1 and cytochrome c, inhibiting the formation of the apoptosome complex (46). Additionally, JNK-mediated phosphorylation of Bad at Thr201 promotes the dissociation of the antiapoptotic protein Bcl-xL from Bad (47). On the other hand, p38 MAPK signaling was implicated in promoting the survival or proliferation of various cancer cell lines, in addition to being associated with poor prognoses in cancer (48,49). The inhibitory phosphorylation of caspase-9 at Thr125 by p38 MAPK was reported to restrain apoptosis (50). The present study revealed that loratadine treatment can suppress activation of p38 and JNK while inducing caspase-8/-9/-3-mediated apoptosis in LUAD cells. Further investigation is warranted to explore the crosstalk between MAPK deactivation and caspase activation in loratadine-induced cell death of LUAD cells.

In addition to MAPKs, STAT3 was identified as another target of loratadine in modulating cell death in LUAD cells. Our findings align with a recent study which demonstrated that cationic amphiphilic antihistamines induce lysosomal H^+^ efflux and cytosolic acidification in cancer cells, subsequently rendering cancer cells more susceptible to apoptosis through STAT3 inactivation. Furthermore, it was observed that a STAT3 inhibitor could sensitize HeLa cancer cells to apoptosis induced by cationic amphiphilic antihistamines (26). To confirm whether the loratadine-induced inhibition of STAT3 in LUAD cells is also linked to lysosomal H^+^ efflux and cytosolic acidification, further validation is required. Additionally, the present study indicated that both a STAT3 inhibitor and JNK and p38 inhibitors could enhance autophagy-mediated apoptosis in LUAD cells. This suggests that not only STAT3 but also JNK and p38 signaling pathways are critical components of loratadine's anticancer mechanisms in LUAD cells.

One of the main serine-threonine phosphatases, PP2A, plays a tumor-suppressive role, as it is often genetically altered or functionally inactivated in numerous solid cancers and leukemia, leading to the promotion of tumor progression through inhibition of PP2A activity (51). Mutations in the genes encoding the regulatory β-subunit of PP2A have also been linked to LUAD proliferation (52). In the present study, it was observed that PP2A was downregulated in LUAD tissues in advanced stages and was correlated with favorable prognoses of patients with LUAD, suggesting that PP2A may play a tumor-suppressive role in modulating LUAD development. Previous research demonstrated that penfluridol, a clinically relevant cationic amphiphilic drug (CAD), can activate PP2A to deactivate the MAPK pathway, resulting in caspase-mediated apoptosis of leukemia cells (53). In the present study, it was also observed that the CAD antihistamine, loratadine, could activate PP2A in LUAD cells, regardless of whether they harbored WT or mutant EGFR. This activation led to the deactivation of downstream signaling pathways such as JNK and p38, ultimately triggering apoptotic cell death. The inhibition of p-STAT3 caused by loratadine was found to be independent of PP2A; however, it was still implicated in loratadine-mediated cell death. It was previously reported that PP2A activation can enhance the sensitivity of chemotherapy drugs in A549 cells with WT EGFR (54) and increase the sensitivity of EGFR tyrosine kinase inhibitors (TKIs) in TKI-resistant LUAD cells with mutant EGFR (55). Vasculogenic mimicry (VM) refers to a novel microvascular structure resembling a three-dimensional channel formed by tumor cells without the involvement of endothelial cells, providing essential nutrients and oxygen to support tumor growth. Research has shown that the presence of VM contributes to increased chemotherapy resistance, distant metastases and poor prognosis in NSCLC. Zhang *et al* (56) demonstrated that activating PP2A plays a crucial role in inhibiting VM formation, as well as reducing invasion and metastasis in NSCLC. In clinical settings, the association between loratadine use and reduced mortality appears to be more pronounced among patients with NSCLC who have records of concurrent chemotherapy compared with those without chemotherapy (22). Therefore, loratadine may hold promise as a potential tool to overcome chemoresistance or TKI-resistance in various LUAD cells by targeting PP2A. In the current study, a preliminary evaluation of the synergistic effects of loratadine was conducted in combination with varying concentrations of the EGFR TKI gefitinib and the chemotherapy drug cisplatin on HCC827 and A549 cells, respectively. Our observations revealed that most combinations did not demonstrate synergistic inhibitory effects on the viability of A549 or HCC827 cells (combination index >1). However, specific combinations, such as 10 *μ*M loratadine with 33 *μ*M cisplatin or 30 *μ*M loratadine with 1 *μ*M gefitinib, exhibited synergistic inhibitory effects (combination index <1) on A549 and HCC827 cells, respectively (data not shown). Further studies are needed to optimize the concentrations of these drug combinations for effective LUAD treatment.

**Figure 8:**
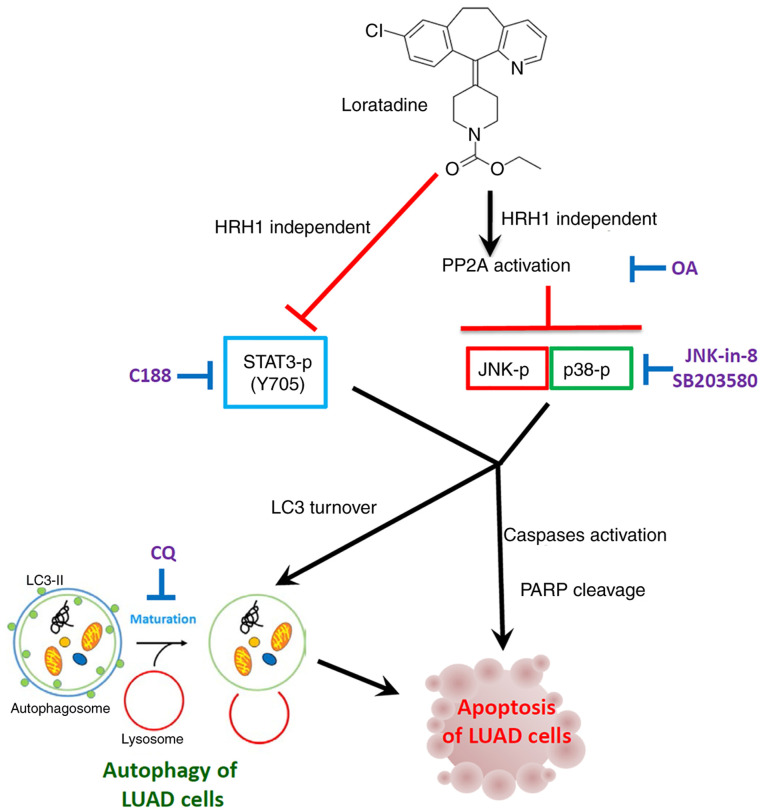
Working model showing the molecular mechanism underlying the ability of loratadine to induce apoptotic cell death of LUAD cells. The anticancer growth effect of loratadine was attributed to its induction of autophagy-mediated-apoptosis independent of HRH1. Mechanistically, loratadine initiates activation of PP2A, leading to the deactivation of p-JNK and p-p38 pathways, ultimately culminating in autophagy-mediated apoptotic cell death. The inhibition of p-STAT3 caused by loratadine is independent of PP2A; nonetheless, it remains implicated in loratadine-mediated cell death. LUAD, lung adenocarcinoma; HRH1, histamine receptor H1; PP2A, protein phosphatase 2A; P-, phosphorylated; JNK, Jun N-terminal kinase; STAT3, signal transducer and activator of transcription 3; CQ, chloroquine; LC3, light chain 3; OA, okadaic acid; PARP, poly (ADP ribose) polymerase.

The present study marks the first time, to the best of our knowledge, that treatment of LUAD cells carrying WT or mutant EGFR with loratadine, a CAD antihistamine, was shown to induce autophagy-mediated apoptosis independent of HRH1. This effect was achieved by triggering PP2A-mediated deactivation of the JNK and p38 signaling pathways. Moreover, STAT3 inhibition was also involved in loratadine-mediated cell death of LUAD. A schematic representation of this mechanism is provided in Fig. 8. Based on these findings, it is proposed to repurpose the safe and cost-effective loratadine for treating LUAD. This repurposing approach may enhance the anti-neoplastic response in combination with chemotherapy or EGFR-targeted therapy, offering a novel therapeutic strategy for LUAD.

## Supplementary Data



## Data Availability

The data generated in the present study are included in the figures and/or tables of this article.
