# Supplementary material for: The off-target effect of loratadine triggers autophagy-mediated apoptosis in lung adenocarcinoma cells by deactivating JNK, p38, and STAT3 signaling through both PP2A-dependent and independent pathways
Source: Int J Mol Med. 2025 Jan 29;55(4):54. doi: 10.3892/ijmm.2025.5495 (PMC11819771; doi:10.3892/ijmm.2025.5495)

Figure S1. (A and B) Knockdown efficiencies of two HRH1 shRNAs were determined by an RT-qPCR in (A) H23 and (B) PC9 cells. (C) HRH1 was overexpressed in A549 cells as determined by an RT-qPCR. Data are presented as the mean ± SD. **P<0.01 and ***P<0.001 vs. the control group. HRH1, histamine receptor H1; shRNA, short hairpin RNA; RT-qPCR, reverse transcription-quantitative PCR.

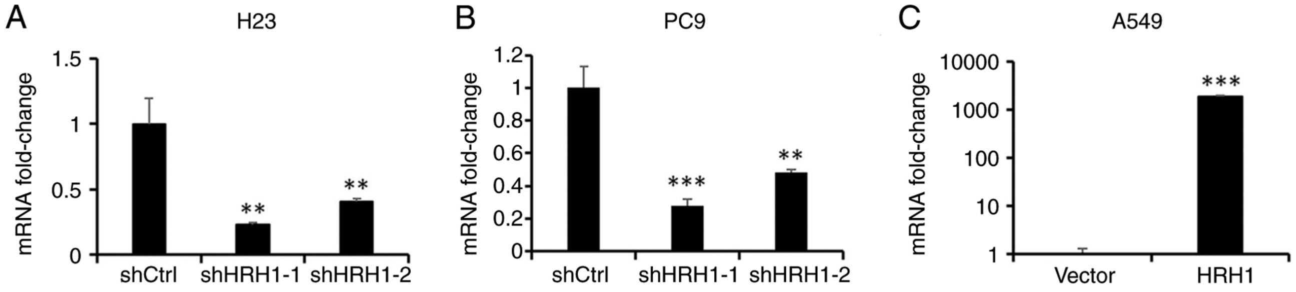

Figure S2. Phosphorylation levels of Akt were evaluated through western blot analysis following a 24-h treatment of H23 or PC9 cells with the indicated concentrations of loratadine. p-, phosphorylated.

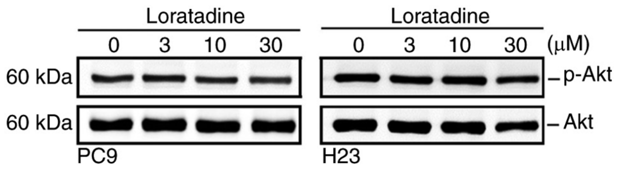

Figure S3. Viability of H23 and PC9 cells treated separately with 10 μM SB203580 (p38 inhibitor), 5 μM JNK-in-8 (JNK inhibitor), 30 μM C188 (STAT3 inhibitor), or 5 nM OA (PP2A inhibitor) for 24 h was evaluated using a Cell Counting Kit-8 assay. The survival rates of inhibitor-treated cells are presented as percentages relative to vehicle-treated cells, which were set as 100%. OA, okadaic acid.

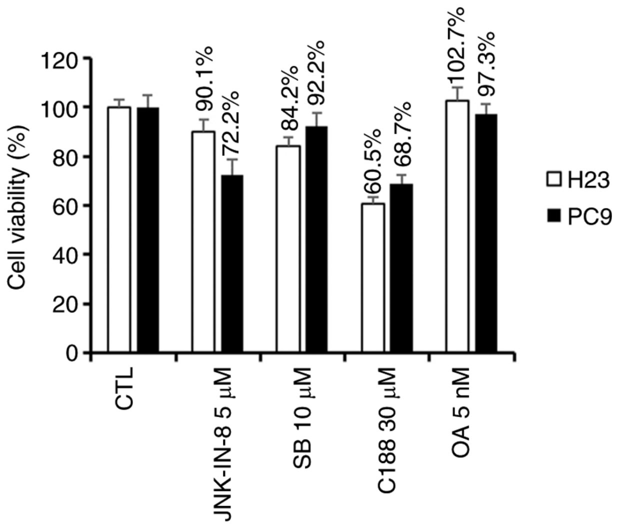

Supplement: Supplementary file 1 [file Supplementary_Data.pdf]
